# Diagnosis and treatment challenges of autism spectrum disorder at a reference hospital in Douala, Cameroon

**DOI:** 10.1186/s12887-023-04242-4

**Published:** 2023-09-13

**Authors:** Daniele Christiane Kedy Koum, Dominique Enyama, Loick Pradel Kojom Foko, Diomede Noukeu, Belviane Nguedia, Rhita Mbono, Charlotte Eposse, Patricia Epee Eboumbou, Cathy Bebey Engome, Yacouba Njankouo Mapoure

**Affiliations:** 1 Faculty of Medicine and Pharmaceutical Sciences, The University of Douala, P.O Box: 8037, Douala, Cameroon; 2 Faculty of Medicine and Pharmaceutical Sciences, University of Dschang, Dschang, Cameroon; 3 Douala Gyneco-Obstetrics and Pediatrics Hospital, Douala, Cameroon; 4 Faculty of Sciences, The University of Douala, Douala, Cameroon

**Keywords:** Autism spectrum disorder, Children, Management, Cameroon

## Abstract

**Background:**

Autism spectrum disorder (ASD) is a neurodevelopmental disability associated with deficiency in social interaction, unusual development of social communication, and restricted or repetitive behaviors, interests and activities. This study aimed to describe management of pediatric ASD in Cameroon, a resource-constrained Central Africa country.

**Methods:**

A retrospective study was conducted between December 2021 and May 2022 at the Pediatrics department of a reference hospital in the town of Douala. Data of interest of children with ASD were collected through eligible medical records and telephone discussions with their parents/guardians.

**Results:**

Medical records of 145 children with ASD aged 2–15 years were included in the study, giving a hospital ASD prevalence of 3.7%. Time delay between parental concerns and hospital management was specified in 69 (47.58%) children, and among them 38 (55.07%) had a mean delay ± SD was less than five months. Children were mainly males (76%) and aged 4–5 years (37.93%), with mean age ± SD of 44.4 ± 22.2 months old. The main consultation reason was delayed language development (100%). Mean time delay between parental concerns and the first medical consultation was 18 months (range 1–60 month). Attention deficit hyperactivity disorder were found in 68.18% of children aged ≥ 6 years old. Neuropsychology (66.2%) was the most frequently used intervention. Some children were treated using traditional medicine.

**Conclusions:**

Management of pediatric ASD is strongly influenced by socioeconomic and cultural context. It is crucial to implement behavioral change campaigns in community, organize training sessions to medical staff on diagnosis and treatment of ASD, and provide specialized centers with skilled staff and equipped material.

## Background

Autism spectrum disorder (ASD) is a neurodevelopmental disability associated with deficiency in social interaction, unusual development of social communication, and restricted or repetitive behaviors, interests and activities [1]. People with ASD usually have isolated activities or can be very intense and focused about a word, conversation or object. Symptoms appear generally before the age of three years, and could manifest during infancy while development is normal during the first year of life [2, 3].

A recent systematic review estimated that the global ASD prevalence ranged ~ 1–4.36% [4]. In The United States of America, the prevalence of ASD was estimated at 2.50% [5]. The burden of ASD in European countries varies between countries with prevalence estimates of 0.48% in France, 3.13% in Island, 0.77% in Finland and 1.26% in Denmark [6]. Epidemiological data on ASD are rare in Africa, and only two studies were available till 2011 across the continent [7]. Seif Eldin and colleagues reported a hospital-based ASD prevalence of 33.6% and 11.5% in Egypt and Tunisia, respectively [8]. In African countries, ASD is poorly known in first-line health caregivers, i.e. nurses and medical doctors, which lead to important diagnosis delays [9].

Deficiencies in social competences – such as restricted interactions, lack of eye contact and emotional reciprocity – are predominantly seen in the most common and severe clinical forms of ASD [10]. Accurate diagnosis of ASD is achievable during the first two years of life [3, 11]. Prompt diagnosis and intervention of ASD are strongly associated with better prognosis [12]. In Cameroon, there is paucity of data on management of ASD which requires a multidisciplinary skilled personnel and adequate technical platform. This study aimed to describe management of ASD in Cameroon, a resource-constrained Central Africa country.

## Methods

### Study design

A retrospective study was conducted between December 2021 and May 2022 at Pediatrics department of the Douala Gyneco-Obstetrics and Pediatrics hospital (DGOPH) in Douala, Littoral Region, Cameroon. The data collection was made on medical record in the retrospective phase and was prospectively completed during phone conversations with parents/guardians.

This hospital has a medical biology laboratory where most examinations for ASD diagnosis are performed (i.e. scanner, auditory evoked potentials, electroencephalograph). The Pediatrics department comports three unities namely (i) a 14-bedded general pediatrics unit dedicated to infants, (ii) a neonatology unit, and (iii) an external consultation unit where neuropediatric consultations are provided, with a medical staff including 3 pediatricians, 1 neuro-pediatrician and 29 medical assistants. Medical consultations of children, whose medical records were included in the study, were performed by pediatricians, and those presenting ASD-evocating signs were referred to the neuro-pediatrician.

### Study population and eligibility

We included medical records of children diagnosed with ASD of both sexes, aged 2–15 years, attending DGOPH from November 2016 to December 2021 (five years), with complete data of interest, and whose parents/guardians given their approval were included in the study. All incomplete medical records were excluded from the study. We also excluded medical records of children whose parents and guardians refused to give information or were not reachable by phone.

The sample size was computed using the Lorentz’s formula N = [p × (1 – p) × Z^2^]/d^2^, where N = sample size required, p = assumed prevalence of ASD, Z = statistic for the desired confidence level (1.96 for 95% confidence level) and d = accepted margin of error (0.05). Based on a systematic review on ASD prevalence [4], the maximum value of prevalence was 4.36%. Thus, the minimum sample size was estimated as N = 64.

### Data collection

Data of each child with ASD were collected through medical records and telephone discussions with their parents/guardians. Data of interest collected during the study were as follows:

- Socio-demographics (name, gender, age on first consultation, delay between first parents’ concerns and first medical consultation);
- Medical history (gestational age, route of delivery, fetal complications, neonatal hospitalization, and familial history of ASD);
- Clinical information (consultation reason, clinical symptoms, warning signs as per the 5th edition of the diagnostic and statistical manual of mental disorders – DSM-5, and comorbidities such as epilepsy, motor/behavior/sleep/food disorders, and attention deficit hyperactivity disorder - ADHD);
- Management of ASD (delay between diagnosis and treatment, type of management).

### Operational definitions

- DSM-5: Fifth edition of the diagnostic and statistical manual of mental disorders.
- Incomplete medical record: Any record with missing clinical information and/or phone number.
- Attention deficit hyperactivity disorder: This comorbidity was diagnosed among children aged ≥ 6 years old as per DSM-5 guidelines.
- Traditional medicine: It consists of resorting to phytotherapy, scarifications and purgative.
- Educative approaches: These consists of several behavioral and developmental approaches to manage children with ASD, and include approaches such as Apply Behavioral Analysis (ABA), Treatment and Education of Autistic and Communication Handicapped Children (TEACCH), and Early Start Denver Model (ESDM).

### Statistical analysis

Data were keyed, coded and verified for consistency in an Excel spreadsheet (Microsoft Office 2016, USA), and then exported to the statistical package for social sciences v20 for Windows (SPSS, IBM Inc., Chicago, Illinois, USA). Qualitative variables were summarized as frequency, percentage, while quantitative variables were presented as mean ± standard deviation (SD).

### Ethical statement

This study was conducted in accordance to national guidelines on animal and human research in vigor in Cameroon. Given the fact that some medical records were incomplete, we contacted parents/guardians of children for complementary information. Medical records of children whose parents/guardians gave their approval and complementary information were retained in the analysis. Confidentiality of data was respected. Finally, research and ethical clearances were issued by ethics committee of the DGOPH (N° 3105 and 2022/0047).

## Results

### Hospital prevalence of ASD

Of the 18,450 children who attended the Pediatrics department of DGOPH for consultation, among them 5,358 received consultation by a neuro-pediatrician. Two hundred were diagnosed with ASD during the study period, giving a hospital ASD prevalence of 1.08% (200/18,450) at Pediatrics ward and 3.7% (200/5,358) at neuro-pediatrics ward. Medical records of fifty-five children were excluded from the study as per exclusion criteria. Thus, medical records of 145 children were finally analyzed in the study.

### Demographics and history of patients with ASD

Socio-demographics and medical history of children with ASD are summarized in Table 1. Males accounted for 76% (110/145) of patients, giving a male-to-female ratio of 3:1. Children were mainly aged 4–5 years (37.93%), with mean age ± SD of 44.4 ± 22.2 months old. Nearly 90% of children were settling in Douala while the rest were living in diverse towns *from* other regions of Cameroon. The mean age ± SD of mothers during their pregnancy was 30.1 ± 3.2 years old, and 53.85% of them were aged 30–35 years. About 23.45% of mothers gave birth by caesarian route.

**Table 1:** Socio-demographics and medical history of children with ASD

Variables	Categories	*n*	%
Gender	Males	110	76
	Females	35	24
Age during consultation (years)	2–3	24	16.55
	4–5	55	37.93
	6–7	40	27.58
	8–11	26	17.93
	12–15	0	0.00
Age at diagnosis (months)	18–35	57	39.31
	36–59	62	42.76
	60–119	24	16.55
	120–180	3	1.37
Term at birth	Term	134	92.41
	Premature	6	4.14
	Post-term	5	3.45

### Clinical characteristics

#### History information

The main consultation reason was delayed language development (100%). More than half of children (53.1%) were received at medical consultation on parental demand. On examination, language disorders (98.6%) were the most frequent signs found in children, followed by impairment or loss of language (70.3%). Mean time delay between concerns and first medical consultations was 18 months (range 1–60 month) with 42.76% of children consulted after 5–10 months (Table 2).

**Table 2:** Anamnestic details of children with ASD

Variables	Categories	*n*	%
Consultation reasons	Delayed language development	145	100
	Behavioral disorder	70	48.3
	Referred to a specialist’s advice	65	44.8
Referent	Otorhinolaryngologist	14	9.7
	Pediatrician	44	30.3
	Physical medicine specialist	10	6.9
	Not addressed	77	53.1
Warning signs	Language disorder	143	98.6
	Loss or impairment of language	102	70.3
	Motor disorders	62	42.8
	Communication disorders	56	38.6
Time delay between first consultation and first parental concerns (months)	< 5	57	39.31
	[5–10[	62	42.76
	[10–24[	24	16.55
	≥ 24	2	1.38

#### Diagnosis of ASD and complementary investigations

Delayed language development was the predominant communication disorders seen in children (96.6%). On analysis of socialization and behavioral domains, 77.9% of children preferred to play alone and 43.4% were either reluctant or cooperative hyperactive (Table 3). Six types of comorbidities were found, and among all children, were greatly represented by behavioral disorder (22.64%). ADHD was found at prevalence of 68.18% (45/66) among children aged ≥ 6 years old. Few children presented more than one comorbidity (Table 4). Neuropsychology (66.2%) was the most frequently medical intervention used to manage ASD. Others complementary investigations were also reported (Table 5).

**Table 3:** Disorders observed by domain of concerns

Variables	Categories	*n*	%
Concerns on communication	Don’t reply with his name	35	24.10
	Don’t know how to ask what he/she wants	46	31.70
	Delayed language development	140	96.60
	Don’t say goodbye	20	13.80
	Don’t respect instructions	36	24.80
	Saying few words before, but nothing now	46	31.70
	Difficulties to hear	29	20.00
Concerns on socialization	Don’t smile	2	1.40
	Poor eye contact	47	32.40
	Prefer play alone	113	77.90
	Don’t want to share objects	31	21.40
	Not interested in other kids	84	57.90
	Ignore everyone around him/her	59	40.70
	Live in our own world	53	36.60
Concerns on behavior	Reluctant or less cooperative hyperactive	63	43.40
	Fit of anger	33	22.80
	Strange movements	44	30.30
	Don’t know how to play with toys	8	5.50
	Hypersensitive to certain sounds/noises	16	11.00

**Table 4:** Comorbidities and complications seen in children with ASD

Comorbidities (n = 106)	*n*	%
Attention deficit hyperactivity disorder	45	42.45*
Behavioral disorder (irritability, aggressiveness)	24	22.64
Epilepsy	13	12.26
Sleep disorders	12	11.32
Food disorders	10	9.43
Motor disorders	2	1.88

*This proportion was calculated by dividing number of children with

ADHD to total number of comorbidities (*n* = 106). The diagnosis of

ADHD is possible at age 6 years old, thus, prevalence of ADHD

was 68.18% among children aged 6 years old (*n* = 66)

**Table 5:** Complementary investigations

Complementary investigations	*n*	%
Neuropsychology	96	66.20
Orthophony	55	37.90
Otorhinolaryngology	48	33.10
Auditory evoked potentials	55	37.90
Electroencephalography	40	27.60
Electroencephalography-based abnormalities	13	9.00

### Management of ASD

Time delay between parental concerns and hospital management was specified in 69 (47.58%) children, among them 38 (55.07%) had a mean delay less than five months. Educative management greatly relied on pedagogy with ordinary education. Rehabilitation based management was mainly performed using neuropsychology. Major drug treatments used were antiepileptic (9%), while a few children were treated with traditional medicines (Tables 5 and 6).

**Table 6:** Delay, drug-free and drug management

Variables	Categories	*n*	%
Time delay between first consultation and first parental concerns (months)	< 5	38	55.07
	[5–10[	20	28.98
	[10–24[	8	11.59
	≥ 24	3	4.34
Rehabilitation approach	ABA	1	0.70
	TEACCH	2	1.40
	ESDM	14	9.70
	Pedagogical management	132	91.00
	Out-of-school	13	9.00
Type of pedagogical management	Ordinary	105	79.54
	Specialized	27	20.45
Type of rehabilitation management	Neuropsychology	49	33.80
	Orthophony	36	24.80
	Psychomotricity	10	6.90
Drug therapies and alternatives	Antiepileptic	13	9.00
	Antipsychotic	5	3.40
	Other drugs	3	2.10
	Traditional medicines	5	3.40
	Potions/Purgatives	5	3.40
	Scarification	2	1.40
	Traditional sorcerer	2	1.40

## Discussion

Prevalence estimate of ASD varies between and within areas [4, 13–16]. A hospital setting-based prevalence was determined in this study, and this could not reflect the real burden of ASD at national level. On average, ASD was diagnosed at the age of 3.5 years with mean delay of medical consultations following identifying of 18-month disorders. This finding is consistent with those of previous studies that reported a long delay in diagnosis and consultations [2, 14, 17–19]. In Africa, cultural beliefs and perceptions are critical to successfully manage diseases such as ASD. Parents consider ASD as a mysterious disease, and this delays greatly medical consultations as they attend hospitals when facing difficulties. In a qualitative study, Mbassi et al. (2012) reported a low level of knowledge and inappropriate attitudes or practices of health caregivers towards ASD [20]. Since 2013, the definition of ASD has been continuously revised, and now ASD includes autism disorder, Asperger’s syndrome, and pervasive developmental disorder not otherwise specified [21]. In developed countries, both diagnosis and consultation of ASD are prompt [11, 18, 22]. Thus, training sessions of medical staff on current diagnosis criteria are needed for early detection and prompt management of ASD in health facilities in developing countries such as Cameroon.

Difficulties in communicating are commonly seen in children with ASD [16, 19, 22–25]. In this study, delayed language development was the main domain observed in children with ASD. In contrast, disturbed social interaction and stereotyped behaviors were less observed. Lotter et al. (1978) pointed out that these two above mentioned signs were less frequent in African countries as compared to Western countries [26]. In this context, parents and health caregivers should be pay attention to communication disorders especially delayed language development for accurate and prompt management of ASD in Cameroon. Additional signs such as deficiency in attention with or without associated hyperactivity should also be taken into account.

The prevalence of ADHD was 68.18% among children aged ≥ 6 years old, and this finding is in line with earlier reports in Kenya [16]. There is a strong genetic, neuropsychological and semiotic overlapping between ASD and ADHD. Indeed, Acquaviva et al. (2014) found that ~ 30–80% of patients with ASD presented diagnostic criteria for ADHD, and conversely ~ 20–50% of patients with ADHD presented diagnostic criteria for ASD [27]. Thus, it seems critical to systematically look for both ASD and ADHD when diagnosis of either of this disorder is confirmed. In addition, ASD goes worse in patients suffering from epilepsy and intellectual deficiency as reported in studies from Nigeria and Tunisia [23, 28]. Bakare et al. (2009) found that intellectual deficiency was the main comorbidity in Nigerian patients with ASD [29].

Of the medical records included in the study, 44.53% of children were managed more than five months after diagnosis. In contrast to this finding, authors reported prompt management of ASD in Morocco [30]. A long delay can install between parental decision making and diagnosis at hospital, which is due to misconceptions related to cultural beliefs.

The bulk of children were attending ordinary education and received education management which mainly relied on pedagogic approach. The literature outlines that children with ASD follow specialized education approaches which are dominated by ABA and TEACHH approaches [31, 32]. In our context, very few schools offer such specialized education approaches to children with ASD. In these schools, fees are often high and are not affordable for parents of children with ASD. Thus, parents are facing difficult choice between keep their children at home and try to send them to high-cost specialized schools. Again, skilled and experienced manpower are dramatically lacking in big towns of Cameroon. Management of children with ASD was majorly performed using neuropsychology, and this does not support previous studies from other settings where orthophony was the main method [30]. The lack of orthophony specialists in our context could likely explain this discrepancy.

Few children received drug treatments, and these were mostly represented by antiepileptic and antipsychotic drugs. This is consistent with findings of Ghita et al. (2015) in Morocco [30]. ASD is still perceived as mystic disease in our context, and parents generally resort to traditional medicine and traditional healers to treat ASD. This could explain why very small fraction of children with ASD received drug treatment in the present study.

### Limitations of study

There were eligible children not included in the study due to several reasons, and this reduced final sample size. The current sample size was not representative of Cameroonian population as the study was conducted in only one health facility, thereby limiting generalization of the present study to population of Cameroonian children with ASD.

## Conclusions

This study aimed at describing management of children with ASD in Douala, Cameroon. ASD was frequently reported at the Pediatrics department of the DGOPH. Communication disorders and ADHD were commonly seen in children. Patients were diagnosed very lately, and this was mainly due to culture-related misconceptions of parents/guardians who majorly resorted to traditional medicines for management of their children with ASD. All these taken together, it is crucial to implement behavioral change campaigns in community, organize training sessions to medical staff on diagnosis and treatment of ASD, and provide specialized centers with skilled staff and equipped material.

## Data Availability

All the data supporting the study findings are within the manuscript. Additional detailed information and raw data will be shared upon request addressed to the corresponding author.
